# Discrimination of SARS-CoV-2 infected patient samples by detection dogs: A proof of concept study

**DOI:** 10.1371/journal.pone.0250158

**Published:** 2021-04-14

**Authors:** Jennifer L. Essler, Sarah A. Kane, Pat Nolan, Elikplim H. Akaho, Amalia Z. Berna, Annemarie DeAngelo, Richard A. Berk, Patricia Kaynaroglu, Victoria L. Plymouth, Ian D. Frank, Susan R. Weiss, Audrey R. Odom John, Cynthia M. Otto

**Affiliations:** 1 Penn Vet Working Dog Center, School of Veterinary Medicine, University of Pennsylvania, Philadelphia, PA, United States of America; 2 Tactical Directional Canine, Smithsburg, MD, United States of America; 3 Department of Pediatrics, The Children’s Hospital of Philadelphia, Perelman School of Medicine, University of Pennsylvania, Philadelphia, PA, United States of America; 4 Department of Criminology, School of Arts and Sciences, University of Pennsylvania, Philadelphia, PA, United States of America; 5 Department of Statistics, The Wharton School, University of Pennsylvania, Philadelphia, PA, United States of America; 6 Department of Medicine, University of Pennsylvania School of Medicine, Philadelphia, PA, United States of America; 7 Department of Microbiology, Penn Center for Research on Coronaviruses and Other Emerging Pathogens, University of Pennsylvania School of Medicine, Philadelphia, PA, United States of America; 8 Department of Clinical Sciences and Advanced Medicine, University of Pennsylvania, Philadelphia, PA, United States of America; University of California-Davis, UNITED STATES

## Abstract

While the world awaits a widely available COVID-19 vaccine, availability of testing is limited in many regions and can be further compounded by shortages of reagents, prolonged processing time and delayed results. One approach to rapid testing is to leverage the volatile organic compound (VOC) signature of SARS-CoV-2 infection. Detection dogs, a biological sensor of VOCs, were utilized to investigate whether SARS-CoV-2 positive urine and saliva patient samples had a unique odor signature. The virus was inactivated in all training samples with either detergent or heat treatment. Using detergent-inactivated urine samples, dogs were initially trained to find samples collected from hospitalized patients confirmed with SARS-CoV-2 infection, while ignoring samples collected from controls. Dogs were then tested on their ability to spontaneously recognize heat-treated urine samples as well as heat-treated saliva from hospitalized SARS-CoV-2 positive patients. Dogs successfully discriminated between infected and uninfected urine samples, regardless of the inactivation protocol, as well as heat-treated saliva samples. Generalization to novel samples was limited, particularly after intensive training with a restricted sample set. A unique odor associated with SARS-CoV-2 infection present in human urine as well as saliva, provides impetus for the development of odor-based screening, either by electronic, chemical, or biological sensing methods. The use of dogs for screening in an operational setting will require training with a large number of novel SARS-CoV-2 positive and confirmed negative samples.

## Introduction

COVID-19, the respiratory infection resulting from SARS-CoV-2 infection, is responsible for the global pandemic starting winter of 2019, with a current infection fatality ratio estimated at 0.5–1% [1, 2]. The disease has spread rapidly as individuals may not show symptoms at the start of the infection or at all, and asymptomatic or presymptomatic carriers can still transmit the disease unknowingly [3]. The rapid spread of the disease has been furthered by limited availability of reagents, low levels of testing and slow turnaround times for results, which makes contact tracing of infected individuals effectively impossible at levels necessary to contain the virus [4]. In order to effectively quarantine infected individuals, cost-effective, rapid, diagnostic tests with high sensitivities (true positive rates) and specificities (true negative rates) are necessary. The most common strategy for diagnosis of new cases of SARS-CoV-2 relies on reverse-transcriptase polymerase chain reactions (RT-PCR) [5]. RT-PCR testing has a high specificity, but false negatives can result from challenges with sampling and false positives may result from the presence of viral RNA without replication-competent virus.

One potential solution for the issues in current testing would be to implement testing based on the volatile organic compound signature (VOC) of SARS-CoV-2 infection. Several diseases have been documented to have unique VOC profiles by either chemical sensors (GC-MS) or biological sensors (dogs) [6–10]. Dogs are able to detect VOC signatures specific to a disease from different biological fluids including urine [11] and saliva [12]. To date, there is strong evidence that there is a unique VOC profile associated with SARS-CoV-2 infection and dogs can be trained to recognize it from saliva/tracheal samples [13] and sweat samples [14]. Jendrny et al. [13] trained eight detection dogs over one week to discriminate between SARS-CoV-2 positive and negative saliva and tracheal secretion samples. Though they presented the dogs with 1012 randomized samples (across all eight dogs), most of these were repeated presentations of samples from the same individuals, despite being initially novel. In the double-blind experiment with seven novel SARS-CoV-2 positive and seven novel SARS-CoV 2 negative samples, though the samples were initially novel, they were presented repeatedly and the data are reported together, making it more difficult to discuss the dogs’ true ability to generalize to completely novel samples. Grandjean et al. [14] trained six detection dogs discriminate between SARS-CoV-2 positive and negative axillary sweat samples, and similarly utilized repeated presentation of samples to the dogs, though they did not find that the success rates were lower on initial presentation of SARS-CoV-2 positive samples (the same information for SARS-CoV-2 negative samples was not included).

The SARS-CoV-2 viral load in biological samples can lead to safety concerns for the personnel and potentially the animals [15, 16] employed for testing [17]. Urine samples tend to have lower viral loads than saliva samples [17], and may represent a relatively safe and easily accessed biological sample. Although these have been shown to have lower viral loads, it is still required to inactivate samples to work with them in the laboratory [18]. It is unknown, however, if dogs can detect the VOC profile in urine and if the virus inactivation method would alter results. If a dog is able to discriminate VOCs in samples associated with SARS-CoV-2 infection from samples from non-infected individuals, then this opens the door to novel screening methodologies based on VOC signatures including electronic noses, chemical signatures (e.g. by gas chromatography and mass spectroscopy) and even using dogs [10]. A VOC-based approach could result in almost instant results where large numbers of people could be screened rapidly and if dogs are utilized, it may be possible to noninvasively and rapidly conduct screenings of large numbers of people.

The aim of this proof of concept study was to determine if dogs trained on detergent-inactivated urine samples from SARS-CoV-2 positive patients could detect heat-inactivated urine samples (sensitivity) from SARS-CoV-2 positive patients and ignore urine samples (either detergent-inactivated or heat-inactivated) from patients that tested negative for SARS-CoV-2 (specificity). A secondary aim was to determine if these dogs trained on urine could detect the presence of SARS-CoV-2 in heat-inactivated saliva samples from hospitalized patients infected with SARS-CoV-2.

## Materials and methods

This study was approved by the University of Pennsylvania Institutional Animal Care and Use Committee for use of Privately Owned dogs (Protocol #806922). The study was approved by the Children’s Hospital of Philadelphia Institutional Review Board for studies involving material derived from human participants (Protocol # IRB 20–071503), as well as the University of Pennsylvania Institutional Review Board for studies involving material derived from human participants (Protocol # 843452). Informed verbal consent was obtained from guardians of participants under the age of 18 and informed written consent was obtained from all adult participants.

### Subjects

We recruited 9 dogs (age range 1.5–6.2, median 1.8 years; 4 male, 5 female; see Table 1) for this study. Dogs were trained with positive reinforcement to show a final trained response (“stand-stare”) at a target odor. The Universal Detector Calibrant (UDC), a synthetic odor impregnated on powder, not found naturally in the environment [19, 20], was used to train the dogs on the mechanics and behavior of searching the scent wheel without utilizing costly and limited patient samples to do so. Through behavioral shaping, when dogs initially approached the container containing UDC, the trainer initiated a conditioned, secondary reinforcer (whistle, clicker or word) to identify the desired behavior and rewarded the dog, with food or a toy. This initial behavior of interest in the UDC was shaped into a 3 second stand-stare behavior (“final response”) at the target odor. No conditioned reinforcer was provided if the dog showed interest in other containers containing control samples or distractors, resulting in extinction of interest in those odors.

**Table 1:** Detection dogs used in this study.

Dog	Breed	Age (years)	Sex	Color
Poncho	Labrador	2.5	M	yellow
Dixie	Labrador	2.4	F	yellow
Tikka	Labrador	1.8	F	black
Miley	Labrador	1.8	F	chocolate
Jake	Labrador	1.5	M	yellow
Blaze	Labrador	1.5	M	black
Tule	Labrador	1.8	F	yellow
M	Labrador	2.2	F	black
Argo	Malinois	6.2	M	sable

### Samples

#### Sample collection

Saliva and urine samples were collected from children (4–18 years of age) hospitalized in the Special Isolation Unit at Children’s Hospital of Philadelphia (CHOP), and adults (18 years or older) hospitalized at the Hospital of the University of Pennsylvania (HUP) who had been diagnosed as SARS-CoV-2 positive using nasopharyngeal swab by a Clinical Laboratory Improvement Amendments (CLIA)-approved RT-PCR test. Negative control samples were obtained from SARS-CoV-2 nasopharyngeal-PCR negative subjects, at the Emergency Department of CHOP for children. Specimens from uninfected adults were collected from asymptomatic, healthy volunteers who had either had a previous negative serologic test for anti-SARS-CoV-2 spike protein IgG and IgM or negative nasopharyngeal swabs. All of the negative volunteers had no symptoms of COVID-19 at the time of collection of the specimens. Exclusion criteria for control subjects included current rhinorrhea, cough, or diarrhea (to exclude individuals with possible false negative SARS-CoV-2 testing). In addition, children, but not adults, were excluded if they required oxygen supplementation within preceding 3 hours of sample collection. Samples were not screened for common circulating non-SARS-CoV-2 human coronaviruses. Patients were not excluded if they had previously tested positive for SARS-CoV-2 or had historical symptoms consistent with SARS-CoV-2. All samples were anonymized.

Urine from all subjects was collected via clean catch into sterile sample cups. Urine cups were provided by nursing staff during routine clinical care. For saliva samples, participants under 18 years of age were requested to tip the cotton ball (CURAD Sterile Cotton Balls, 1” or 2.54 cm) into their mouth without touching it with their hands. Clinical/study staff assisted subjects during saliva collection when necessary. Participants were instructed to hold the cotton ball between the lower lip and teeth for approximately 60 seconds, spit the cotton ball back into the plastic container, and replace the container lid. For adult patients, saliva was collected by having individuals spit into a sterile collection cup.

In addition, control urine samples were obtained from the Perelman Medical Center at the University of Pennsylvania’s Biobank. These were control samples from a clinical trial that were collected in 2010, pre-COVID-19, and had been frozen at -80 until processing.

#### Sample preparation and storage

Urine: To inactivate any viable virus in the samples, two different treatments were applied: 1) detergent NP-40 (Thermo Scientific^TM^ Nonidet^TM^ P40 Substitute) [21] and 2) heat inactivation based on recommendations of the University of Pennsylvania Environmental Health and Safety Office. Eleven samples from children (Table 2) and two samples from adults (Table 3) were treated with 1% NP-40. All remaining samples were heat-treated as shown in Tables 2 and 3. For the children’s urine samples, an aliquot of 1 mL was transferred into a 10 mL glass vial which was then heated to 56°C for 60 minutes in a dry block heater. Urine samples from adults were incubated for five minutes at 95°C. All samples were processed in a biosafety level-2 (BLS-2) laboratory biosafety cabinet. After sample inactivation, samples were stored at a minimum of -20°C cold storage until aliquoting.

**Table 2:** Children’s demographic and clinical characteristics for urine and saliva samples.

Variables	SARS-CoV-2 negative (*n* = 14)	SARS-CoV-2 positive (*n* = 11)	*p* value
**Demographic characteristics**			
Age (years), median (IQR)	15 (11.5–16)	11 (9–17)	0.26
Female, *n* (%)	8 (57.1)	6 (54.5)	>0.99
Black or African-American, *n* (%)	6 (43)	6 (55)	0.70
**Reported Symptoms, n (%)**			
Fever (>38.0°C)	0 (0)	5 (45.4)	0.009
Cough (new onset or worsening of chronic cough)	0 (0)	4 (36)	0.026
Sore throat	0 (0)	1 (9)	0.44
Headache	0 (0)	1 (9)	0.44
**Samples collected, n (%)**			
NP-40 urine	6 (43)	5 (45)	
Heat treated urine	7 (50)^1^	6 (54)	
Heat treated saliva	13 (93)	9 (82)	

Data are median value (interquartile range) or number of patients (%).

^1^ One SARS-CoV-2 negative patient had previously tested positive, one had not been previously tested but reported to have had signs consistent with COVID-19 previously (this information was not available initially).

**Table 3:** Adult demographic and clinical characteristics for urine and saliva samples.

Variables	SARS-CoV-2 negative (*n* = 4)	SARS-CoV-2 positive (*n* = 5)	*p* value
**Demographic characteristics**			
Age (years), median (IQR)	40 (29, 58)	46 (34, 54)	0.94
Female, *n* (%)	2 (50)	1 (20)	0.52
Black or African-American, *n* (%)	1 (25)	4 (80)	0.21
**Reported Symptoms, n (%)**			
Fever (>38.0°C)	0 (0)	4 (80)	0.05
Cough (new onset or worsening of chronic cough)	0 (0)	3 (60)	0.17
Muscle Aches	0 (0)	2 (40)	0.44
Headache	0 (0)	1 (20)	>.99
Nasal oxygen	0 (0)	2 (40)	0.44
**Samples collected, n (%)**			
NP-40 urine	0 (0)	2 (40)	
Heat treated urine	4 (100)	4^1^ (80)	
Heat treated saliva	4 (100)	5 (100)	

Data are median value (interquartile range) or number of patients (%).

^1^ Two urine samples were divided and half NP-40 treated and half heat treated, one patient urine sample was not available

Saliva: the cotton ball (children) was placed into a 10 mL vial and heat inactivated at 95°C for 5 minutes. Saliva from adults was inactivated using same protocols. After inactivation, saliva samples were diluted 1:5 in sterile 0.9% sodium chloride (B.Braun Medical, Irvine CA) to decrease viscosity and allow aliquoting. All inactivated saliva samples were aliquoted, in a BLS-2 laboratory biosafety cabinet.

After sample inactivation, all samples were stored at a minimum of -20°C cold storage until aliquoting. Samples from adults or children were combined, but only NP-40-inactivated urine was mixed with NP-40-inactivated urine, heat-inactivated urine was only mixed with heat-inactivated urine and heat-inactivated saliva was only mixed with heat-inactivated saliva. Samples were aliquoted into 400 uL volumes into SciK9 Training Aid Delivery Devices (TADD) [22]. Samples included individual patients (400 uL) or combinations from 2 patients (200 uL each), 3 patients (133 uL each) or 4 patients (100 uL each). Though it is likely that mixing samples of multiple patients does not result in multiple novel samples, these mixes were utilized in an attempt to present the dogs with a higher number of unique odor profiles due to the low number of completely novel samples available. See Table 2 for demographic and clinical characteristics of children’s samples and see Table 3 for demographic and clinical characteristics of adult samples. Except for age, Fisher’s Exact test was used for contingency table analysis. For age, unpaired t-tests were used.

### Training

**Fig 1:**
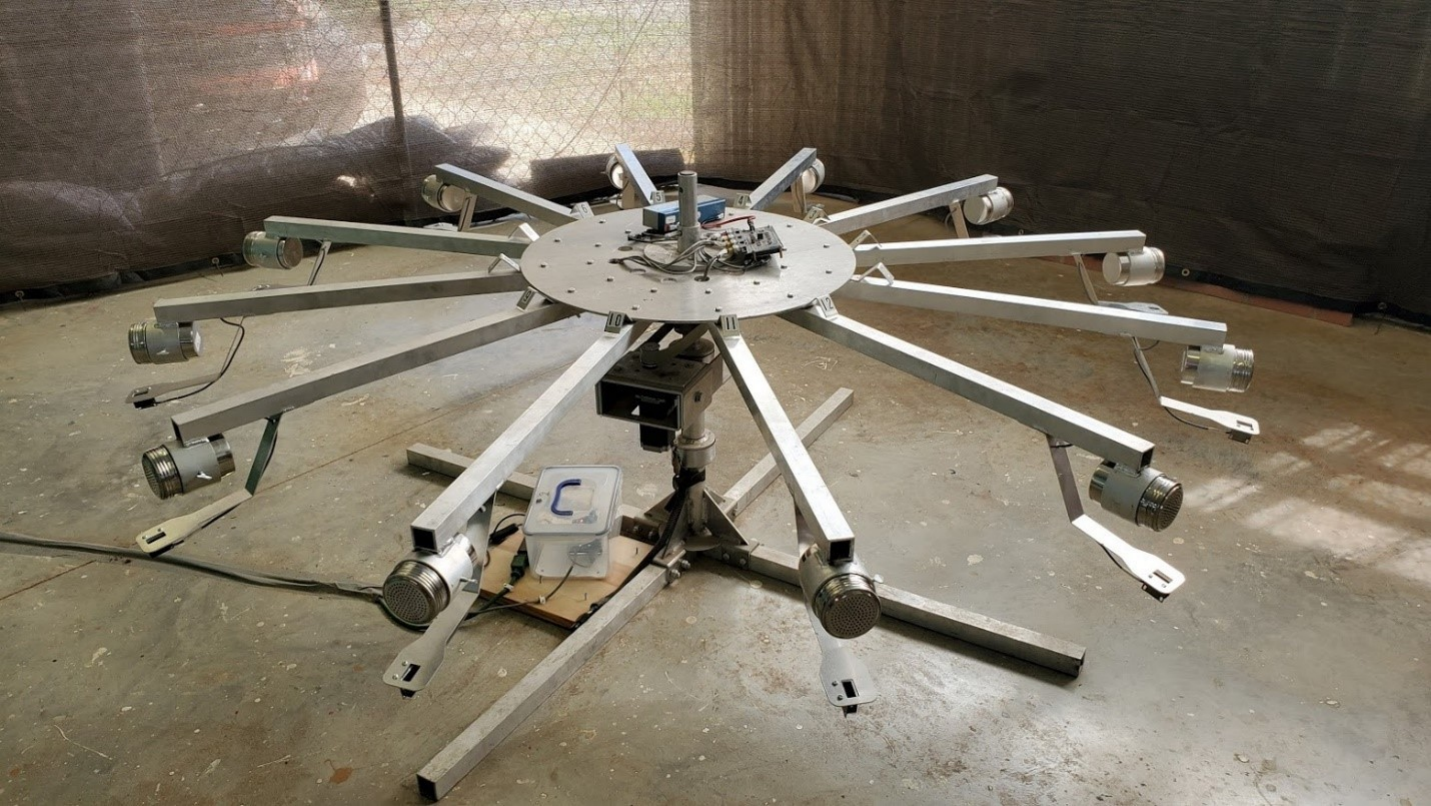
Scent wheel used in this study.

**Fig 2:**
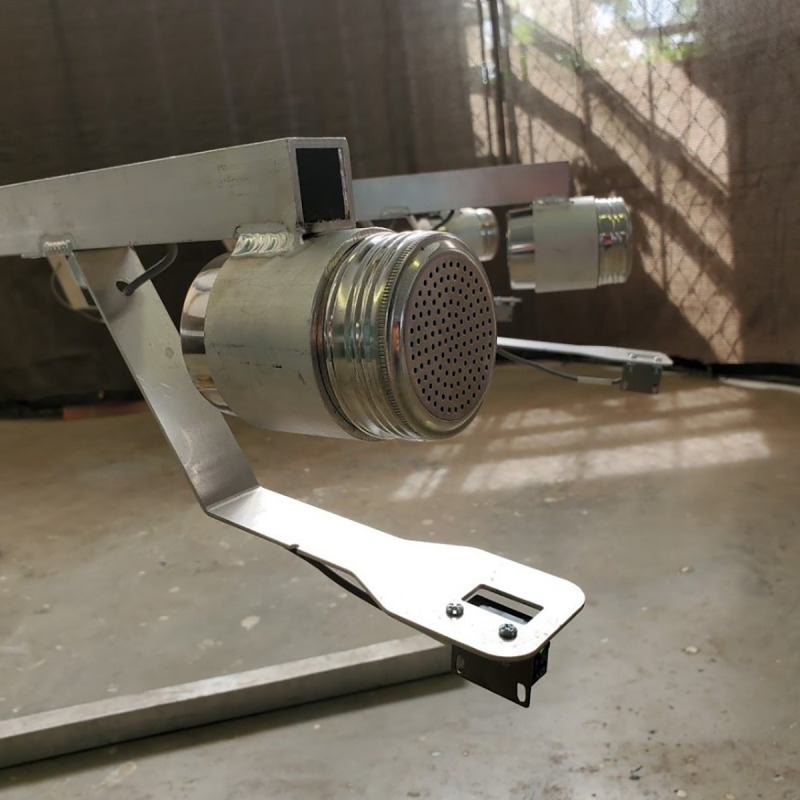
Close up of port on scent wheel.

For the purposes of this study, dogs were trained to systematically search 12-port scent wheels for their target odor (initially UDC) (Fig 1). These scent wheels had 12 arms with removable stainless-steel ports on the end (Fig 2), such that not only the target odor but also its holder could be cleaned and/or replaced between trials. Each port was monitored by a motion sensor which recorded the duration of time the dog spent at the port. Each wheel contained controls (e.g. SARS-CoV-2 negative samples treated with the same inactivation protocol as targets) as well as distractors (e.g. gloves, empty TADD, garlic on filter paper, Sharpie^TM^ marker on filter paper, TADD with coconut flavoring, paperclips, marinade on filter paper, empty cans etc), such that each port contained an odor.

In training, the dogs were exposed to two scenarios: one where the scent wheel contained their target odor in one port and control or distractor odors in the other ports, and one where the scent wheel contained all control or distractor odors. The scent wheel containing all non-target odors, also known as a “blank wheel”, was introduced to the dogs so that they would not view the task as a forced-choice task, as has been suggested to be suboptimal in medical detection settings [23]. Initially, trainers were in-sight of the dogs while they searched the scent wheel, but over time were moved to an out-of-sight scenario and watched the dogs through a window in the dividing screen. When the scent wheel contained the dog’s target odor, and the dog showed their trained final response (alert) at the target odor, as in training, the trainer pressed a clicker and rewarded the dog. When the scent wheel was blank, the dogs were allowed to search for 2 complete rotations and then were called out of the scent wheel area. If at any point during training, the dog showed interested in or exhibited a trained final response on a non-target odor, the behavior was ignored, unless the dog persisted for more than 3 seconds and then the dog was given the verbal no reward marker “no”.

After being trained to search for odor with UDC, the dogs were trained to find urine from SARS-CoV-2 positive patients which was treated with NP-40, while ignoring both NP-40 treated urine from SARS-CoV-2 negative patients and NP-40 in water. Similar to the initial training on the UDC, dogs were presented with a sample containing 400 uL of urine from SARS-CoV-2 positive patient on a single stand and when dogs sniffed the container, the trainer used a conditioned secondary reinforcer (a whistle, or pressed a clicker that beeped and initiated an automated release of food as a reward for the dog). This behavior was shaped into their 3 second stand-stare trained final response. Initially, the target urine sample in the single port was the same as (matched to) the target urine sample on the wheel, so the dogs were finding the same target odor amongst controls and distractors. After three- and one-half weeks of training, this procedure was changed. The urine sample in the single stand was changed from a matching sample to a unique target odor, so the dogs were required to identify the common odor in the samples on the stand and on the wheel.

During initial training, dogs were presented with 38 unique combinations of NP-40 treated targets from 7 SARS-CoV-2 positive individuals (5 children, 2 adults) and 24 unique combinations of NP-40 treated targets from 6 SARS-CoV-2 negative individuals (all children).

Presentation of the SARS-CoV-2 negative controls followed the design of errorless learning applied to odor detection training with early introduction of the nontarget odor [24, 25]. Initially, the SARS-CoV-2 negative controls were presented at lower levels than the SARS-CoV-2 positive samples, in order to initially give the dogs two elements to utilize to distinguish the positive samples from controls (e.g. positive vs. negative and more vs. less). The control urine samples were introduced with a single hole drilled in the lid of the TADD to limit odor dissipation. As dogs continued in training without interest in the control samples, the number of holes in the lids were increased until the lids were removed at two- and one-half weeks of training and the odor dissipation was equivalent to that of the targets.

Training was conducted 5 days a week, with dogs exposed to 2–5 scent wheels daily. Newly processed samples were provided weekly as they became available. Once the dogs reached greater than 90% accuracy in training, different testing scenarios were presented in order to determine to what extent this initial training on NP-40 treated urine from SARS-CoV-2 positive patients would allow the dogs to generalize to other types of biological samples from SARS-CoV-2 positive patients.

## Results

We present here the results of the dogs’ accuracy which is based on the training where the dogs could explore the wheel in any direction and make more than one revolution before being called away, as well as sensitivity (the true positive rate divided by true positives plus false negatives for finding the sample on the first pass of the port), specificity (the number of true negatives divided by the number of true negatives plus the number of false positives).

Confidence intervals were constructed cognizant of the repeated measure design for which there were several trials for each dog. The full dataset was split into two, random, disjoint subsets, a training dataset (N = 183), and a test dataset (N = 182). Using the training data separately for accuracy and false “hits”, a lasso regression analysis was applied using the dog’s name as a set of eight indicator variables. After regularization, none of these indicators were selected. This result was confirmed by a conventional logistic regression using the test data. The null hypothesis: there was no association between each dog indicator variable and either outcome variable, was not rejected. Consequently, a confidence interval from the test data was constructed separately for both response variables, ignoring each dog’s identity, using 1000 randomly permuted versions of the outcome variable. The 95% confidence interval for both was approximately ± 0.12. The 95% confidence interval for a conventional, less robust approach was approximately ± 0.10. These apply to each dog’s performance. The confidence interval over all dogs was approximately ± 0.04. The differences result from the number of observations for each dog compared to the number of observations for all dogs at once.

### NP-40-treated urine to heat-treated urine

For the four days prior to introduction of the heat-treated urine sample, the dogs trained on detergent-treated urine searched a total of 14 wheels, each with 4 control samples and one target, and the remaining ports held distractors. The dogs showed an overall accuracy rate of 94%. The overall sensitivity was 71% and the overall specificity was 99%. To determine if this initial training would allow the dogs to also recognize heat-treated SARS-CoV-2 positive urine, dogs were tested on two separate trials, one in which they were presented with first a known detergent-treated urine sample and sent to find a heat-treated SARS-CoV-2 positive urine sample from the same individual, and one in which they were presented with a novel heat-treated SARS-CoV-2 positive urine sample and then sent to find a second novel heat-treated SARS-CoV-2 positive urine sample. In the first trial, SARS-CoV-2 negative urine controls were detergent-treated while in the second trial, they were heat-treated. Eight dogs completed the two trials. On the first trial, all dogs alerted on the target sample, two on the first pass, and two exhibited a false alert on non-target samples. For the second trial, five dogs alerted to the target on the first pass, two dogs exhibited a false alert on non-target samples, and one dog never alerted to the target sample. The odor profile the dogs used to discriminate between SARS-CoV-2 positive and negative urine samples seemed to be maintained between these two types of inactivation, at least enough that dogs trained on detergent-treated were able to transfer readily to heat-treated inactivation. After evidence of recognition in the heat-treated urine samples, the dogs then continued training on the heat-treated urine.

### Novel mixes of known heat-treated urine

**Fig 3:**
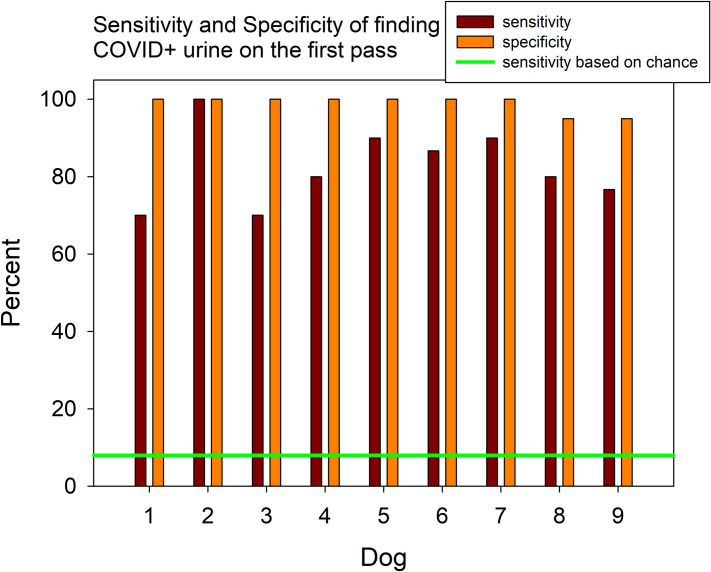
**Sensitivity (dark red bars) and specificity (orange bars) of the individual dog’s response to novel mixes of familiar SARS-CoV-2 positive heat-treated urine samples on the first time they encountered the odor (first pass).** The green line represents sensitivity based on chance.

For the four days prior to testing on heat-treated urine, the dogs searched a total of 14 wheels, two wheels had three controls and one target, and the remaining 12 had four control samples and one target. The overall sensitivity was 71% with an accuracy rate of 94%. The specificity was 98%. Dogs were tested on five trials of novel mixes of known heat-treated SARS-CoV-2 positive samples. At the beginning of a trial, the dog was presented with one novel mix of known heat-treated SARS-CoV-2 positive samples, then the dog was sent to search a scent wheel which contained a novel mix of known heat-treated SARS-CoV-2 positive samples in one of the ports, while other ports contained novel mixes of known heat-treated SARS-CoV-2 negative samples (four per trial) and distractors. Nine dogs completed the five trials. The accuracy rate for identifying the target was 96%, based on the training where the dogs could explore the wheel in any direction and make more than one revolution before being called away. Overall sensitivity (the true positive rate divided by true positives plus false negatives for finding the sample on the first pass of the port) was 68%, while the overall specificity, (the number of true negatives divided by the number of true negatives plus the number of false positives) was 99% (Fig 3). The false positive rate was 1%.

### Urine to saliva

Prior to testing whether training on SARS-CoV-2 positive urine would allow the dogs to recognize SARS-CoV-2 positive saliva, the dogs were exposed to control SARS-CoV-2 negative saliva samples in the wheel with SARS-CoV-2 positive urine samples, where they exhibited only two false alerts out of 72 exposures. Dogs were then tested on two separate trials, similar to the protocol used when testing heat-treated urine after training on NP-40-treated urine. The dogs were first presented with a known heat-treated SARS-CoV-2 positive urine sample and then sent to find a SARS-CoV-2 positive saliva sample from the same individual. In the second trial, they were presented with a novel SARS-CoV-2 positive saliva sample and then sent to find a second novel SARS-CoV-2 positive saliva sample. In each trial, the other ports contained four novel SARS-CoV-2 negative saliva samples and seven distractors. Nine dogs completed the two trials. In the first trial, there were no false alerts on control samples, while six dogs gave a trained final response, two on first pass, three after the second pass, and one after a third pass. One dog did not give a trained final response but did show a change of behavior at the target, suggesting some level of recognition or odor similarity to the trained target, and two dogs failed to alert on any sample. In the second trial, two dogs gave a false alert, but both subsequently alerted on the target sample, one dog alerted on initial contact with the target, three dogs alerted on second pass and three dogs showed a change of behavior without a trained final response at the target. Thus, all dogs trained to discriminate positive and negative SARS-CoV-2 patient urine samples showed recognition in these patients’ saliva samples as well.

### Novel heat-treated urine

To test for dogs’ ability to generalize to novel heat-treated SARS-CoV-2 positive samples and ignore novel heat-treated SARS-CoV-2 negative samples, dogs were tested in a second setup where they were not presented with any samples that had been previously trained as individual samples or as part of mixtures. Dogs were tested over three trials, and there was no odor presented before being sent to search in the scent wheel. In each of the first and second trials, there was one novel heat-treated SARS-CoV-2 positive sample, four novel heat-treated SARS-CoV-2 negative samples, seven controls. In the third trial, dogs were presented with a blank wheel, with four novel heat-treated SARS-CoV-2 negative samples and eight distractors. Nine dogs completed the five trials. All dogs initially exhibited a false alert on at least one control sample on each wheel. Two dogs eventually gave a trained final response on the target odor. Four dogs, after being called off the control and passing the target once, had a change of behavior at the target that would be consistent with a level of recognition, but not a clear response.

Over all trials (i.e., all training conditions for all dogs, N = 365), accuracy was 92.5% except for this novel heat-treated urine test condition where the accuracy was 11.1%. The effect size was to reduce accuracy by 81.4 percentage points. Using logistic regression, it is not surprising that the p-value for rejecting the usual null hypothesis is well below 0.0001. Results for all dogs are shown in Table 4.

**Table 4:** Total number of positive samples presented, negative samples presented, sensitivity and specificity (first pass on odor), as well as accuracy, across dogs for teach training and testing scenario.

	Positive samples	Negative samples	Sensitivity	Specificity	Accuracy (%)
**Training (n = 9 dogs)**					
NP-40 Treated	14	56	71	99	94
Heat Inactivated	14	54	71	98	94
**NP-40 to Heat Inactivated Test (n = 8 dogs)**					
Trial 1 (first exposure)	1	4 (NP-40)	75	98	100
Trial 2 (novel)	1	4 (Heat)	62	98	62
Blind test of Heat Inactivated (n = 9* dogs)					
Novel mixes	5	20	68	99	96
Novel samples	2	12	18	41	11**
**Urine to Saliva (n = 9 dogs)**					
Trial 1 (first exposure)	1	4	22	100	67
Trial 2 (novel)	1	4	11	94	100**

* For one dog, Trial 2 of Novel Samples is missing.

** The accuracy includes “change of behavior”, if the dog showed interest in the odor but did not exhibit a full final trained response (“alert”).

## Discussion

Though dogs have previously been shown to be able to discriminate between saliva samples of SARS-CoV-2 positive and negative patients [19], these studies are also using repeated presentations of the same samples. Thus, it is possible that the result is simply that dogs are able to discriminate between their training set of positive and negative patient samples but are unable to generalize this odor to new samples. Initially, in our study, the introduction of completely novel samples did not appear to pose a problem, however, after a period of intensive training on a limited number of samples, the dogs appeared to move from generalization strategies to more discrimination of individuals in the training set. Despite mixing samples to increase the number of sample odor profiles that the dogs were exposed to in an attempt to support their generalization of a SARS-CoV-2 odor profile for positive samples, in our testing scenarios, we were only able to document that our dogs were able to general to novel mixes of known SARS-CoV-2 positive and negative samples, and were unable to generalize to completely novel samples. It is unknown how the dogs would have responded, if blinded testing with novel samples was conducted prior to the period of intensive training with the same or related samples. It is possible that reducing the number of presentations that dogs had on identical training odors may have facilitated their generalization, however without further investigation the question remains unanswered.

An additional complication came from two samples that were initially identified as SARS-CoV-2 negative samples, both of which were presented to the dogs as a control. These two samples (and their subsequent mixes) resulted in consistent alerts by the majority of the dogs. One sample was from an individual who, though their RT-PCR test result at the time of entry into this study was SARS-CoV-2 negative, they had recently recovered from COVID-19, during which time they were SARS-CoV-2 positive. The second sample was from an individual who had recent COVID-19 symptoms (at one point prior to testing negative for their sample in this study), although was RT-PCR negative upon enrolment in this study. Due to the methods used in this study to mix samples to create novel odor profiles, the dogs were presented with samples from these patients across many presentations and continued to “falsely” identify presumed control samples before these samples were removed from the training set. The use of these samples resulted in confusion across all dogs that had to be mitigated through further training, as well as decreased the number of available control samples the dogs could be presented with through training. This may have affected the dogs’ abilities to generalize the odor, both by reducing the number of samples they could learn to discriminate, as well as confusing them for a period of time before we removed the samples from their training set. One unexpected benefit of this complication, however, is that the ability of the dogs to recognize these novel samples, suggests that the dogs were generalizing to some degree. Since, there is no gold standard to confirm that the odor profile of these two “controls” was the same as the SARS-CoV-2 positive samples, this interpretation cannot be verified. It does, however, reiterate the point raised by Edwards et al [23], that confirmed negative samples are critical. Grandjean et al. [14] had a similar issue, with two of their supposed SARS-CoV-2 negative controls turned out to be positive, once they contacted the hospitals after the dogs signalled that they believed the samples were positive. With SARS-CoV-2, the false negative test rate of RT-PCR testing [11] and unknown duration of a signature odor after recovering from COVID-19, further complicate the procurement of control samples.

Although these results support the presence of a VOC profile associated with COVID19, they raise important questions when training dogs to learn a COVID-19 odor profile. The ideal number of samples, the diversity of patients providing samples, and the extent of training that dogs need to generalize to novel samples in this odor problem remains unknown. The training utilized in this study did not result in documented generalization of a SARS-CoV-2 positive odor profile, despite dogs showing impressive discrimination between positive and negative samples. This suggests that either the number of samples, or the number of sample presentations, though likely both, need to be better suited for not just discrimination but also generalization (see S1 File for specifics on sample usage). Future training of dogs and investigation into biological, chemical and electronic detectors should focus on increasing the number of relevant and novel samples. Dogs are already being deployed for real-time detection of SARS-CoV-2 positive individuals [26]. Because dogs will readily respond to the stimulus that is rewarded most frequently, this generalization is critical for potential deployment of dogs in the search for a rapid diagnostic test for COVID-19. The more novel profiles of both positive and confirmed negative samples without repetition of individuals will promote generalization by the dogs and identification by the sensors of the specific COVID-19 target odor profile.

## Supporting information

S1 File (PDF)
