# Supplementary material for: Discrimination of SARS-CoV-2 infected patient samples by detection dogs: A proof of concept study
Source: PLoS One. 2021 Apr 14;16(4):e0250158. doi: 10.1371/journal.pone.0250158 (PMC8046346; doi:10.1371/journal.pone.0250158)
Supplement: S1 File — (PDF) [file pone.0250158.s001.pdf]

Supplemental Information

Sample usage

Initial training on NP-40 treated urine involved 38 unique COVID19 positive samples, mixed from 7 patients. Samples were aliquoted into 400 uL volumes into SciK9 Training Aid Delivery Devices (TADD)[S1]. Samples included individual patients (400 uL) or combinations from 2 patients (200 uL each), 3 patients (133 uL each) or 4 patients (100 uL each), to expand the number of novel odor profiles available. The individual samples were used for both the initial stand and on the scent wheel. On the initial stand with 19 exposures, samples were used a median of 1 time each (IQR 1.0, 1.2); on the scent wheel, with 62 exposures, target SARS-COV-2 positive samples were used a median of 2 times each (IQR 1.0, 2.5); overall the dogs were exposed to the same sample 2.1 +/-1.2 times.

Training on heat-treated urine involved 34 unique COVID19 positive samples, mixed from 10 patients (2 of which also had NP-40 treated samples). The individual samples were used for both the initial stand and on the scent wheel. On the initial stand with 44 exposures, samples were used a median of 2 times each (IQR 1.0, 2.0); on the scent wheel, with 84 exposures, target SARS-COV-2 positive samples were used a median of 3 times each (IQR 1.0, 4.0); overall the dogs were exposed to the same sample a median of 5 times each (IQR 3.0, 6.0). To further complicate the training picture, prior to the blinded testing on the heat-treated urine, the training involved 29 unique mixes; whereas in the 2 weeks between the heat-treated urine testing and the testing of completely novel samples, the dogs were only exposed to 12 of those mixes. Prior to the heat-treated blinded test, dogs were exposed to samples a median of 3 times, (IQR 2.0, 4.0) each, after the blinded test, dogs were exposed to samples a mean of 2.6 +/- 1.3 times, however the cumulative exposure to those samples was 5.3 +/- 1.4 times. To further complicate, the training, those samples represented only 6 individuals.

Samples from control patients represented an additional challenge. Of the 24 unique combinations for NP-40 treated urine, they were generated from 6 patients. Of the 51 unique combinations of heat-treated urine, 20 generated from 8 patients were only used for the novel testing, leaving 31 for training, generated from 8 patients. In that group, one patient was later discovered to have previously tested positive for SARS-COV-2 and another had reported symptoms of COVID-19 but no definitive test prior to testing negative at study entry. The dogs consistently alerted to the samples that were from these patients or mixes that contained samples from these patients. Based on the history, these samples were removed from the training mix, leaving only 16 control samples for training.

References:

S1. Maughan MN. Methods of using training aid delivery devices (TADD). (2017) Available at: https://patents.google.com/patent/US20170367298A1/en [Accessed August 14, 2020]
